# Clinical significance and risk factors of incidental parathyroidectomy after total thyroidectomy

**DOI:** 10.1007/s12020-025-04225-8

**Published:** 2025-04-09

**Authors:** Mehmet Kostek, Isik Cetinoglu, Zerin Sengul, Hazal Arikan, Mehmet Taner Unlu, Ozan Caliskan, Nurcihan Aygun, Mehmet Uludag

**Affiliations:** 1 Division of Endocrine Surgery, Department of General Surgery, University of Health Sciences Sisli Hamidiye Etfal Training and Research Hospital, Istanbul, Turkey; 2 Department of General Surgery, University of Health Sciences Umraniye Training and Research Hospital, Istanbul, Turkey

**Keywords:** Total thyroidectomy, Hypoparathyroidism, Hypocalcemia, Parathyroid, Central neck dissection

## Abstract

**Purpose:**

Postoperative hypoparathyroidism is the most common complication after total thyroidectomy(TT). The clinical significance of incidental parathyroid glands(IP) detected in pathological examination of removed surgical material is still controversial. The aim of this study was to evaluate the clinical follow-up findings and risk factors of patients with IP.

**Methods:**

Postoperative pathology results and postoperative biochemical findings of patients who underwent TT±Central Neck Dissection(CND)/Lateral Neck Dissection(LND) between September,2020 and September,2023 in single institution were retrospectively evaluated. Patients with IP were divided into Group-1, and patients without IP were divided into Group-2. Patients of Group-1 were divided as Single IP and Double IP subgroups according to the number of IP.

**Results:**

The findings of a total of 412 patients were evaluated. IP was detected in 90(21.8%) of the patients. Postoperative hypoparathyroidism was more common in Group-1 at the 6th hour, 24th hour and 1st month postoperatively (69.7% vs. 31.7%, *p* < 0.0001; 61.1% vs. 27.7%, *p* < 0.0001, 26.2% vs. 12.2%, *p* = 0.002, respectively). Persistent hypoparathyroidism was observed at a rate of 5.3% in Group-2 and 11.5% in Group-1 (*p* = 0.041). Postoperative hypocalcemia was seen more frequently in Group-1 than in Group-2 at the 12th hour (34.4% vs. 23.2%, *p* = 0.031, respectively). There was no difference between Single IP vs. Double IP groups for the serum levels of Calcium and PTH. Among the risk factors evaluated for the detection of IP, in the univariance analysis, operation indications(*p* = 0.018), CND(*p* < 0.0001), surgeon experience(*p* = 0.016), thyroid gland volume(*p* = 0.02), preoperative serum TSH value(*p* = 0.031); in multivariance analysis, operation type ( ± CND) (OR:2.785; 95% CI: 1.175–6.605; *p* = 0.020) and operator experience between 10–20 years (OR: 0.117, 95% CI: 0.033–0.418, *p* = 0.001) and >20 years (OR: 0.254, 95%CI: 0.085–0.760, *p* = 0.014) were found significant compared to operators experienced <5 years.

**Conclusion:**

Patients with detected IP after total thyroidectomy have lower postoperative Calcium and PTH. Significant risk factors for the IP were low level of surgeon experience and undergoing CND.

## Introduction

Postoperative hypoparathyroidism is the most common complication after total thyroidectomy (TT) [1]. After thyroid surgery, the rate of this complication varies from 14 to 60% of patients [2, 3].Postoperative hypoparathyroidism causes hypocalcemia and postoperative hypocalcemia leads to significant decrease in quality of life and increases healthcare costs [4, 5]. Treatment of hypocalcemia includes use of oral calcium replacements, and these medications trigger gastric intolerance, abdominal distention, constipation or bloating [6].

Possible explanations for hypoparathyroidism are impaired arterial or venous blood flow and surgical removal of nonpathological parathyroid glands (incidental parathyroidectomy[IP]). Meticulous surgical dissection and increased attention for preserving parathyroid glands are necessary for prevention postoperative hypoparathyroidism. However, parathyroid glands can be resected unintentionally even in experienced hands, especially when it is intrathyroidal or subcapsular [7, 8]. Also the appearance of parathyroid glands could be confused with the lymph nodes, fat or thyroid nodules [9]. New techniques such as near infrared autofluorescence can help identification and preservation of parathyroid glands and their vascular pedicle. However, there is not enough study for evaluating their efficacy for decreasing postoperative hypoparathyroidism [10].

The clinical significance of incidental parathyroid glands encountered in pathology reports in the postoperative period is still controversial [11]. The relationship between IP and postoperative hypocalcemia and hypoparathyroidism was investigated in several studies, however, there is no consensus between these studies [12–19]. Due to the low possibility of postoperative hypocalcemia and hypoparathyroidism in patients who underwent thyroid lobectomy, the clinical effect of IP is more evident in patients with total thyroidectomy [20]. Total thyroidectomy is a surgical procedure which is mostly performed to treat Graves’ Disease, Toxic or Nontoxic Multinodular Goiter and Thyroid Malignancies. Due to the dissection of both sides of the thyroid gland, the risk of injury to the parathyroid gland is increased. In addition to the total thyroidectomy, Autoimmune Thyroiditis may cause fibrosis in thyroid gland in which preservation of parathyroid glands could be more difficult [21]. Also, Lymph node metastasis can be seen in thyroid malignancies and dissection of central neck lymph nodes may potentially harm parathyroid glands as well as it can lead to unintended resection of parathyroid glands [22]. In order to prevent IP during total thyroidectomy, risk factors which play role for IP should be well understood.

The aim of this study is to evaluate the clinical significance and risk factors of incidental parathyroid glands detected in postoperative pathology reports.

## Materials and methods

**Fig. 1:**
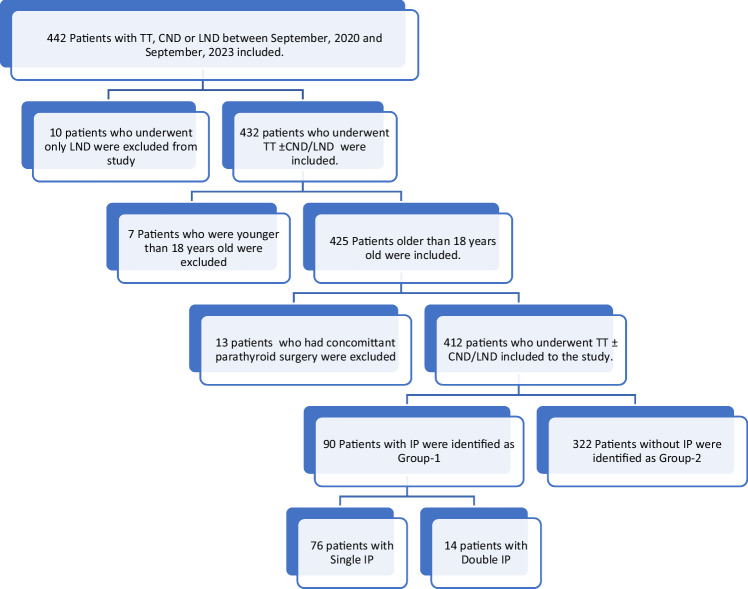
Selection flowchart for the patient population. TT total thyroidectomy, CND central neck dissection, LND lateral neck dissection

Patients who underwent TT ± Central Neck Dissection(CND)/Lateral Neck Dissection(LND) between September, 2020 and September, 2023 in the Department of General Surgery were evaluated retrospectively. Demographic information, past medical history, preoperative imaging and biochemical findings, preoperative diagnosis, indications for surgery, intraoperative findings and final diagnosis, presence and the number of IP in the postoperative pathology report as well as postoperative biochemical findings were collected. CND was performed in the patient group who had pathologically confirmed central or lateral neck metastasis (therapeutic CND), in the patients with differentiated thyroid cancer bigger than 4 cm, with extrathyroidal extension or with medullary thyroid cancer (prophylactic CND). LND was performed in patients with pathologically confirmed lateral neck metastasis. Patients underwent planned parathyroidectomy in addition to TT, patients underwent only CND and/or LND and patients younger than 18 years old were excluded from the study. A flowchart which gives the detail of patient selection is illustrated in Fig. 1. All patients who underwent thyroid surgery had preoperative ultrasound examinations which were performed by the same endocrine radiologist with a 5- to 14-MHz linear transducer (Aplio 500; Canon Medical Systems, Tokyo, Japan).

In order to evaluate the clinical effects of IP, patients with an IP seen in the removed surgical material were included to the Group-1, and patients without IP were included to the Group-2. Laboratory findings and the presence of postoperative hypoparathyroidism and hypocalcemia were compared between the two groups. Serum Calcium and PTH values were measured in postoperative 6th,12th, 24th hour, 1st and 6th month and in postoperative 6th, 24th hour, 1st and 6th month, respectively. Reference level of hospital laboratory for Serum Calcium was between 8.6–10.2 mg/dl and for Serum PTH was between 16–65 ng/dl. Postoperatively, Serum Calcium levels below 8 mg/dl were considered as biochemical hypocalcemia, and Serum Parathormone (PTH) levels below 15 ng/dl were considered as hypoparathyroidism. In patients with PTH level were still below 15 ng/dl at the end of 6 months were considered as persistent hypoparathyroidism [2].

In patients with serum PTH levels between 10 and 15 ng/dl in postoperative 6th hour, Calcium Citrate 2500 mg twice daily were started. PTH levels less than 10 ng/dl in postoperative 6th hour, Calcitriol 0.5 mcg twice daily and Calcium citrate 2500 mg twice daily were started and titrated until serum calcium levels were in normal range. These medications are weaned off according to serum PTH levels in postoperative follow-up visits. Also, in symptomatic patients with serum PTH levels > 15 ng/dl, Calcium Citrate 2500 mg twice daily were started.

In order to determine their role as risk factors for IP, patient age, gender, operation indication (Malignancy, Suspicion of Malignancy, Thyrotoxicosis, Benign), operation type (TT ± CND ± LND), secondary surgery(previous history of subtotal thyroidectomy), surgeon experience (years in practice<5 years, 5–10 years, 10–20 years and >20 years), total thyroid volume(measured in Ultrasonography), largest nodule diameter, presence of substernal thyroid gland, total operative time, presence of nodules in both sides of thyroid gland, preoperative L-thyroxine use and preoperative TSH level were compared between the two groups. Statistically significant risk factors were included to multivariate analysis to better understand their effect on IP.

### Statistical analysis

Compliance of the data with normal distribution was evaluated with the Shapiro-Wilk test. Mean and standard deviation were used for normally distributed variables, median and interquartile ranges were used for non-normally distributed variables. Chi-square (χ2) and Fisher’s exact test were used to compare categorical data. In comparisons between groups, Student t-test and Mann Whitney U test were used for parametric and nonparametric data, respectively. Statistical significance level was accepted as *p* < 0.05. Parameters that are significant in univariate analysis were included in multivariate analysis with binary logistic regression model. Results were considered as statistically significant when p-value less than 0.05. Statistical analysis was performed using Statistical Package for Social Sciences (SPSS) version 25.0 (IBM Co., Armonk, NY, USA).

### Ethics statement

This study was performed in line with the principles of the Declaration of Helsinki. Approval was granted by the Local Ethics Committee of our institution (Date: 25.06.2024, Number: 4450). This study was reported according to the Strengthening the Reporting of Observational Studies in Epidemiology (STROBE) guidelines [23].

## Results

The findings of a total of 412 patients were evaluated. The number of male patients was 88 (21.4%) and 324 (78.6%) were female. The average age was 48.22 ± 13.16 years. IP was detected in pathological examination in 90 (21.8%) of the patients. Single IP was detected in 76 patients (84.4%), and double IP was detected in 14 (15.6%) patients. Intrathyroidal IP were detected in 3 patients(3.3% of IPs and 0.72% of all patients). Parathyroid autotransplantation was applied in 34 (8.2%) patients. The number of the surgeons who were responsible for the thyroid surgeries were distributed according to their experience as 3 surgeons less than 5 years, 3 surgeons between 5–10 years, 1 surgeon between 10–20 years and 1 surgeon more than 20 years.

Postoperative 24th hour, 1st and 6th month serum PTH levels and postoperative 6th, 12th, 24th hour,1st and 6th month serum calcium levels were significantly lower in Group-1 compared to Group-2(Table 1). There was no difference for postoperative 6th hour Ca levels between two groups.

**Table 1:** Mean and standard deviation of postoperative calcium and PTH results.

	Group-1 (*n*:90)			Group-2 (*n*:322)	*p*-values				
	Single IP	Double IP	All Patients in Group-1		Single IP vs. Double IP	Single IP vs. Group-2	Double IP vs. Group-2	Double IP vs. Single IP vs. Group-2	Group-1 vs. Group-2
6th h Ca	8.69 ± 0.56	8.38 ± 0.50	8.64 ± 0.56	8.74 ± 0.46	0.085	1.00	***0.020***	**0.021**	0.09
6th h PTH	15.28 ± 12.60	8.10 ± 7.94	14.14 ± 12.23	25.94 ± 20.19	0.567	***<0.001***	***0.002***	**<0.001**	***<0.001***
12th h Ca	8.24 ± 0.62	7.92 ± 0.65	8.19 ± 0.63	8.40 ± 0.59	0.197	*0.101*	***0.010***	**0.002**	***0.003***
24th h Ca	8.24 ± 0.59	8.06 ± 0.68	8.21 ± 0.60	8.42 ± 0.63	0.975	0.069	0.105	**0.012**	***0.005***
24th h PTH	15.28 ± 12.53	9.06 ± 7.22	14.31 ± 12.05	27.85 ± 20.12	0.755	***<0.001***	***0.001***	**<0.001**	***<0.001***
1st month Ca	9.16 ± 0.74	8.75 ± 0.91	9.09 ± 0.78	9.38 ± 0.60	0.082	***0.033***	***0.001***	**<0.001**	***0.002***
1st month PTH	31.69 ± 22.39	28.56 ± 13.88	31.17 ± 21.17	42.15 ± 32.33	1.00	***0.029***	0.305	**0.013**	***0.004***
6th month Ca	9.08 ± 0.77	8.62 ± 0.98	9.00 ± 0.83	9.33 ± 0.58	0.085	***0.033***	***0.001***	**<0.001**	***0.004***
6th month PTH	34.58 ± 19.32	32.48 ± 18.56	34.23 ± 19.07	42.09 ± 22.38	1.00	0.074	0.460	**0.040**	***0.012***

Bold and italic values represent statistically significant p values (*p* < 0.05)

**Fig. 2:**
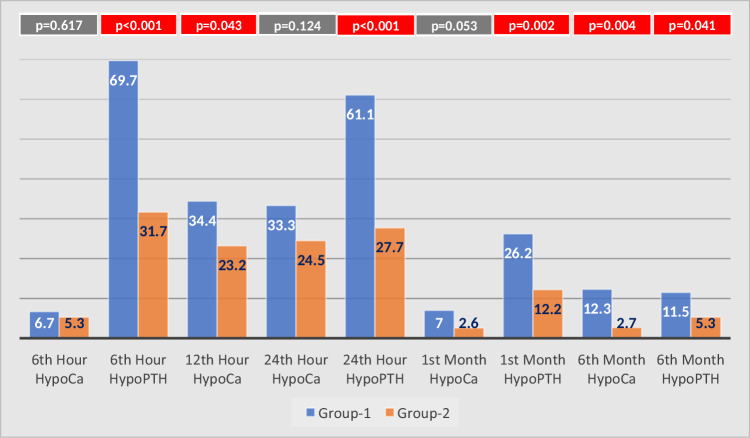
The rates of hypocalcemia(HypoCa) and hypoparathyroidism(HypoPTH) in postoperative 6th, 12th, 24th h, 1st and 6th month for the patient population

Postoperative hypoparathyroidism was significantly more common in Group-1 at the 6th hour, 24th hour and 1st month postoperatively (69.7% vs. 31.7%, *p* < 0.0001; 61.1% vs. 27.7%, *p* < 0.0001, respectively; 26.2% vs. 12.2%; *p* = 0.002). Persistent hypoparathyroidism was observed at a rate of 5.3% in Group-2 and 11.5% in Group-1 (*p* = 0.041). Postoperative hypocalcemia was seen significantly more frequently in Group-1 than in Group-2 at the 12th postoperative hour (34.4% vs. 23.2%, respectively, *p* = 0.031) (Fig. 2).

**Fig. 3:**
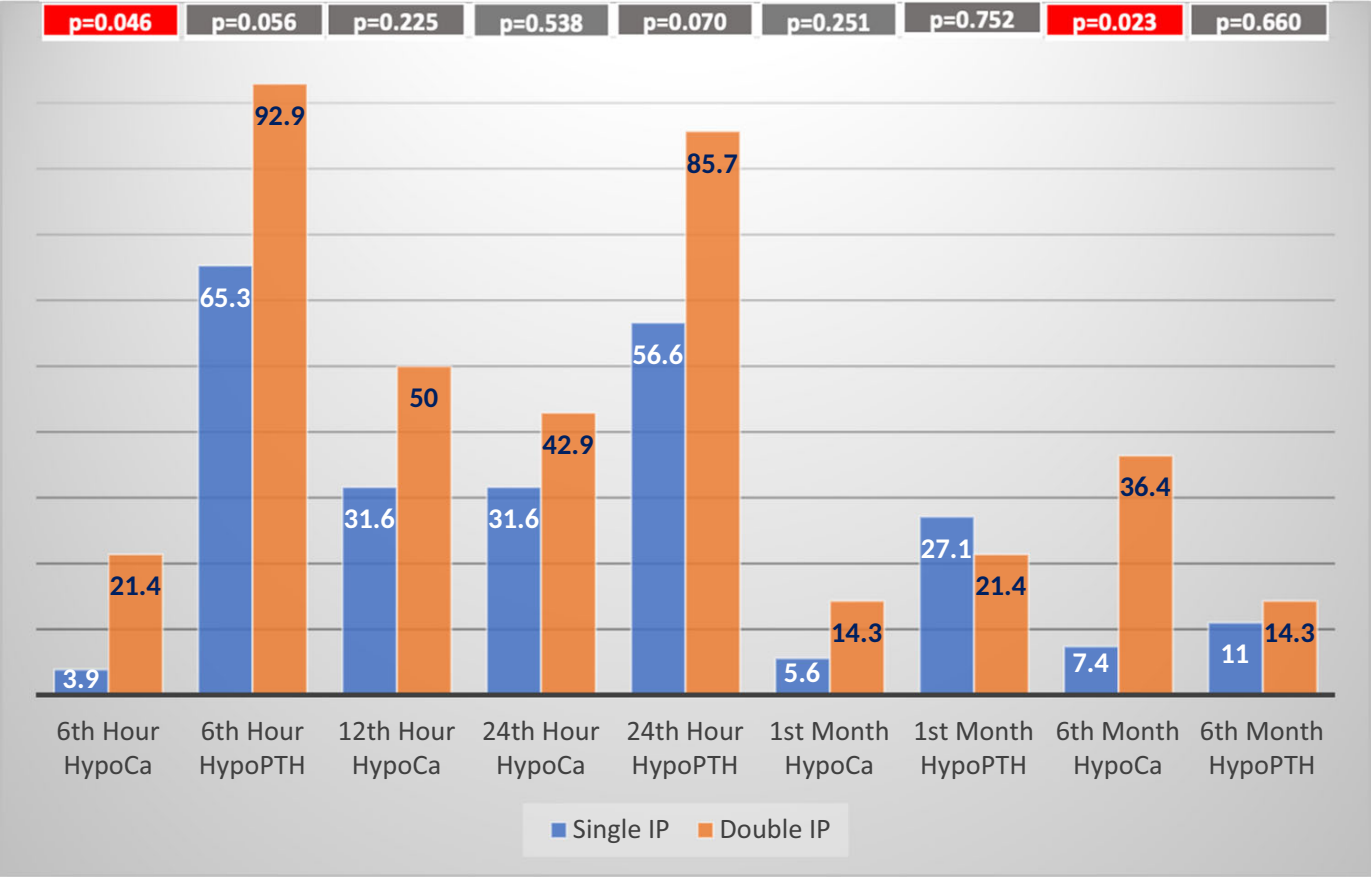
The rates of hypocalcemia(HypoCa) and hypoparathyroidism(HypoPTH) in postoperative 6th, 12th, 24th h, 1st and 6th month for the patients with incidental parathyroidectomy IP incidental parathyroidectomy

The rates of hypocalcemia and hypoparathyroidism were compared between patients with single IP or double IP which were found out in pathological investigation. The rate of postoperative hypoparathyroidism in the double IP group was higher at 6th, 24th hour and 6th month postoperatively, however, the results were not statistically significant. On the other hand, the rate of postoperative hypocalcemia in the double IP group was significantly higher in 6th hour and 6th month postoperatively. The rate of postoperative hypocalcemia in the Double IP group was also higher in the 12th and 24th hour and 1st month postoperatively, however, the results were not significant statistically(Fig. 3). The postoperative levels of serum Calcium and PTH were also compared between single IP and double IP group, however, there was no difference between two groups(Table 1).

Among the risk factors evaluated for the detection of IP, in the univariance analysis, operation indications (*p* = 0.018), operation type (±CND) (*p* < 0.0001), operator experience (*p* = 0.016), thyroid gland volume (*p* = 0.02), preoperative serum TSH value (*p* = 0.031) were found to be significant (Table 2). Statistically significant parameters were included to the multivariance analysis. In the multivariance analysis operation type (+CND) (OR: 2.785; 95% CI: 1.175–6.605; *p* = 0.020) and operator experience between 10–20 years and >20 years were found significant compared to operators experienced <5 years (OR: 0.117 and 0.254, 95% CI: 0.033–0.418 and 0.085–0.760; *p* = 0.001 and 0.014, respectively) (Table 3).

**Table 2:** Univariate analysis of the risk factors for incidental parathyroidectomy.

Univariate analysis		Group-1(*n*:90)	Group-2(n:322)	*p*-value
Age (years)		48.2 ± 13.2	48.7 ± 12.7	0.769
Gender	Female	71 (78.9%)	253 (78.6%)	0.948
	Male	19 (21.1%)	69 (21.4%)	
Operation indication	Malignancy	31 (34.4%)	61 (18.9%)	***0.018***
	Suspicion of Malignancy	8 (8.9%)	29 (9%)	
	Thyrotoxicosis	18 (20%)	87 (27%)	
	Benign	33 (36.7%)	145 (45%)	
Operation type	TT	67 (74.4%)	290 (90.1%)	***<0.001***
	TT+CND±LND	23 (25.6%)	32 (9.9%)	
Secondary surgery	Yes	7 (7.8%)	19 (5.9%)	0.687
	No	83 (92.2%)	303 (94.1%)	
Surgeon experience	<5 years	7 (41.2%)	10 (58.8%)	***0.016***
	5–10 years	48 (24.5%)	148 (75.5%)	
	10–20 years	7 (10.0%)	63 (90.0%)	
	>20 years	28 (21.7%)	101 (78.3%)	
Total thyroid gland volume (cm^3^)		54.4 ± 76.1	62.6 ± 75.5	***0.02***
Largest nodule diameter (mm)		24.6 ± 13.9	27.6 ± 16.4	0.125
Substernal thyroid gland	Yes	16 (17.8%)	54 (16.8%)	0.831
	No	74 (82.2%)	268 (83.2%)	
Presence of nodules in both sides of thyroid gland	Yes	63 (76.8%)	249 (85.3%)	0.07
	No	27 (23.2%)	73 (14.7%)	
Total operative time (min)		166.6 ± 76.2	153.8 ± 56.4	0.142
Preoperative L-thyroxine use	Yes	16 (17.8%)	42 (13%)	0.254
	No	74 (82.2%)	280 (87%)	
Preoperative serum TSH levels (mIU/L)		1.69 ± 1.63	1.63 ± 3.67	***0.031***

*TT* total thyroidectomy, *CND* central neck dissection, *LND* lateral neck dissection, *TSH* thyroic stimulating hormone

Bold and italic values represent statistically significant p values (*p* < 0.05).

**Table 3:** Multivariate analysis of the risk factors for incidental parathyroidectomy.

Multivariate analysis		Odds ratio (95% CI)	*p*-values	
Preoperative serum TSH levels		0.996 (0.914–1.085)	0.929	
Total thyroid gland volume		1.000 (0.997–1.004)	0.831	
Operation indication	Benign	1 (reference)	0.657	
	Malignancy	1.543 (0.649–3.664)		0.326
	Suspicion of malignancy	1.377 (0.541–3.504)		0.502
	Thyrotoxicosis	0.903 (0.465–1.753)		0.763
Operation type	TT	1 (reference)		
	TT+CND±LND	2.785 (1.175–6.605)	***0.020***	
Surgeon experience	<5 years	1 (reference)	***0.004***	
	5–10 years	0.411 (0.146–1.157)		0.092
	10–20 years	0.117 (0.033–0.418)		***0.001***
	>20 years	0.254 (0.085–0.760)		***0.014***

*TT* total thyroidectomy, *CND* central neck dissection, *LND* lateral neck dissection, *TSH* thyroic stimulating hormone

Bold and italic values represent statistically significant p values (*p* < 0.05).

## Discussion

Mortality and morbidity of thyroidectomy have been improved since the beginning of 20th century [24]. However, postoperative hypoparathyroidism and hypocalcemia are still most common complications after thyroidectomy [25]. The aim in this study was to understand the impact of IP to the development of postoperative hypoparathyroidism and hypocalcemia and risk factors of IP. In order to understand the effect of IP, only patients underwent TT were included to the study. Due to the one-sided dissection of thyroid gland during lobectomy, parathyroid glands on the contralateral side are not going to be dissected, and this may decrease the rate of IP or parathyroid glands which are not dissected may hide the clinical effect of IP by compensating hormonal activity of the excised gland. During TT, both sides of the thyroid glands as well as all four parathyroid glands were dissected. The dissection of four parathyroid glands in all patients helps to have more homogenous population in the study group and the clinical effects of IP are more evident.

In this current study, the rates of hypoparathyroidism were 39.1% at the 6th hour and 34.9% at the 24th hour postoperatively. When Group-1 and Group-2 were compared for postoperative hypoparathyroidism rates, there were significantly higher rate of hypoparathyroidism in the Group-1 at early postoperative period (6th hour, 24th hour), first month and 6th month. Similar to our findings, the rate of hypoparathyroidism in the previous studies varies between 14–60% of patients [2, 3, 26]. Additionally, the rate of IP was 21.8% in our patient population. In the previous studies, the rate of IP has been shown between 2.3 and 24.9% [27]. However, these wide variations mostly occur due to the different inclusion criteria in each study. Consequently, the rates of hypoparathyroidism and IP in our study are consistent with the previous studies in the literature.

The effects of IP on postoperative hypocalcemia and hypoparathyroidism were studied previously, however, there is no consensus on this topic. In several previous studies, there was no proven relationship between IP and hypocalcemia. However, in these studies, patients underwent thyroid lobectomy were included to the studies. Due to the low rate of hypoparathyroidism and hypocalcemia in thyroid lobectomy patients, there was no statistically significant relationship between IP and postoperative hypocalcemia or hypoparathyroidism [12–14]. A significant relationship between IP and postoperative hypocalcemia or hypoparathyroidism were shown in various studies consistent with findings of this study [15–19].

According to the recent studies, the rate of permanent hypoparathyroidism was 0–14.5% in the literature. In this study, the rate of permanent hypoparathyroidism was 6.5% in total patient population. Exclusion of lobectomy patients from this study and the variations between studies for the criteria of hypoparathyroidism may be increased the rate of permanent hypoparathyroidism [28–31].

The rate of postoperative hypoparathyroidism and hypocalcemia was evaluated in patients with single or double IP. Comparison between these two groups was shown higher rate of postoperative hypoparathyroidism in 6th and 24th hour and 6th month postoperatively in the double IP group, however, results were not significant due to the very low number of patients with double IP. Also, the rate of postoperative hypocalcemia in the double IP group was significantly higher in 6th hour and 6th month. The rate of postoperative hypocalcemia in the double IP group was higher in the 12th and 24th hour and 1st month postoperatively but these results were not statistically significant. Moreover, serum levels of Calcium and PTH were similar between Single IP and Double IP group, postoperatively. In a previous study, postoperative 24th hour calcium and PTH results of single or double IP were compared, however, there was no difference between two groups which is also consistent with our findings [32]. The reasons for the insignificant results could be the low number of double IP patients and the use of calcium and calcitriol replacement for postoperative hypoparathyroidism.

Serum PTH levels were measured at the 6th hour postoperatively and calcium replacement in addition to calcitriol were started if PTH < 10 pg/mL. Treatment for prevention of hypocalcemia was started depending on early measurement of serum PTH level. Therefore, the number of symptomatic patients due to hypocalcemia was very low and wasn’t included to the statistical analysis.

Surgical removal and vascular injury to the parathyroid glands are the two main mechanisms for hypoparathyroidism. CND is an advanced surgical procedure which requires extra care to preserve the parathyroid glands and their vascular structures [33]. In this study, performing CND were shown to increase the risk of IP approximately 2.8 times compared to only performing TT (95% CI:1.175–6.605). Furthermore, several studies were shown that CND is a risk factor for IP [14, 34, 35].

Resection of intrathyroidal parathyroid glands together with thyroid gland may be another reason for hypoparathyroidism. In this study, the rate of intrathyroidal parathyroid glands was 0.72% and was similar to the cadaveric studies [36]. However, there are variability about the rates of intrathyroidal parathyroid glands different in clinical studies. In previous studies, the rate of intrathyroidal parathyroid gland was between 1.7–11.0% [12, 17, 37, 38]. The possible reasons of this variability between numbers are uncertainty of the criteria for intrathyroidal parathyroid gland, discrepancy of pathological evaluation of thyroid gland between different hospitals and inconsistent sensitivity for locating intrathyroidal parathyroid glands.

Previous studies were shown the importance of surgeon experience for the outcomes of thyroid surgery [39, 40]. Also, American Thyroid Association Guidelines recommends experienced surgeons to manage complicated cases for avoiding complications [3]. In this current study, it was shown that increased surgeon experience decreases the IP rate(*p* = 0.004). Patients operated by surgeons experienced less than 5 years had higher rate of IP than patients operated by surgeons experienced between 5–10 years, however, it was not statistically different(41.2% vs. 24.5%, OR: 0.411, 95%CI: 0.146–1.157, *p* = 0.092, respectively). Furthermore, surgeons experienced 10–20 years or more than 20 year showed lower rate of IP compared to the surgeons experienced <5 years (10.0% vs. 41.2%, OR: 0.117, 95% CI: 0.033–0.418, *p* = 0.001; 21.7% vs 41.2%, OR:0.254, 95%CI: 0.085–0.760, *p* = 0.014, respectively).

Most experienced surgeons more likely to manage more complicated cases and it may cause slightly increased rate of IP, but still significantly low from less-experienced surgeons. Various studies on risk factors of IP were shown no impact of surgical experience on IP. In a previous study, the rates of IP were evaluated between consultant and registrar surgeons and there was no statistically significant difference for IP between two patient group (19.06% vs. 15.88%, *p* = 0.40, respectively) [41].In another study, the rates of IP for residents, attending surgeons, associate professor and professors were compared, however, there was no statistical difference for the rates of IP (10.7%, 9.1%, 13.1%, 0%, *p* = 0.253, respectively) [38]. These results may be related to inclusion criteria of the studies.

Contrarily, in a recent study, the impact of surgeon volume on IP was studied and higher surgeon volume for thyroidectomy were shown to decrease IP rate(R^2^ = 0.77, *p* = 0.008). The IP rate in this study was 22.4% (range: 16.9–43.6%) [42]. In a previous multi-center study, decreased rate of hypoparathyroidism were shown with increased surgical experience(*p* = 0.02). Surgeons between 5–20 years of experience showed best performance for prevention of hypoparathyroidism. However, this study were not mentioned IP in this patient group [43]. Along with these studies, our study showed that surgeon experience is important for decreasing complications of thyroidectomy such as IP and hypoparathyroidism.

In addition to more dedicated approach for understanding the effect of IP, new technologies can be used to prevent IP such as near infrared autofluorescence techniques [44]. Indocyanine Green Angiography (ICGA) for parathyroid glands may help to understand parathyroid gland vascularization and viability after dissection of glands. ICGA can prevent both inadvertent resection of parathyroid gland and harming parathyroid vascular structures unintentionally [45].Additionally, after resection of thyroid gland, autofluorescence techniques may help finding accidentally resected parathyroid glands on the resected thyroid gland and allows auto transplantation of resected parathyroid gland [46]. Further studies are needed to show the efficacy of new techniques for prevention of IP.

The main limitations of this study are retrospective design and postoperative use of calcium and calcitriol. Patients with low PTH values were started calcium replacement and calcitriol in postoperative 6th hour. Serum calcium and PTH levels in the follow-up visits may be affected by calcium and calcitriol replacement. Another limitation of this study is evaluation of postoperative materials by different pathologists. In future prospective studies for understanding the effect of IP for postoperative hypoparathyroidism and hypocalcemia, pathological examination should be done by single pathologist by using a specific criteria and surgical findings during the operation should be noted to define observed parathyroid glands.

As a conclusion, IP significantly affects postoperative serum Calcium and PTH levels and plays important role for postoperative hypocalcemia and hypoparathyroidism. The significant clinical risk factors for IP are low level of surgeon experience and CND. In patients with incidental parathyroid glands detected in the pathological examination after total thyroidectomy±CND should be closely monitored for hypoparathyroidism and hypocalcemia in the postoperative follow-up, and it should not be forgotten that they may be at higher risk for persistent hypoparathyroidism. In patients who undergo CND with TT, importance should be given to preserving the parathyroid glands, and less-experienced surgeons should pay attention to preserve parathyroid glands.

## Data Availability

No datasets were generated or analysed during the current study.
